# Effects of Different Pretreatments on Wheat Bran and Its Arabinoxylan Obtained by Sequential Extraction with Dilute Alkali and Alkali–Urea Mixture

**DOI:** 10.3390/foods14040696

**Published:** 2025-02-18

**Authors:** Axiang Liu, Shengjie Zhang, Wentao Wang, Hanxue Hou, Yangyong Dai, Cheng Li, Hui Zhang

**Affiliations:** 1 College of Food Science and Engineering, Shandong Agricultural University, Tai’an 271018, China; liulax1999@163.com (A.L.); zsj410502@163.com (S.Z.); wangwt@sdau.edu.cn (W.W.); hhx@sdau.edu.cn (H.H.); dyyww@sdau.edu.cn (Y.D.); 2 Engineering and Technology Center for Grain Processing of Shandong Province, Tai’an 271018, China; 3 Department of Food Science and Nutrition, Culinary Institute, University of Jinan, Jinan 250022, China

**Keywords:** cell wall, hemicellulose, ferulic acid, defatting, deproteinization, delignification, antioxidant capacity

## Abstract

Arabinoxylan (AX), an abundant and highly valuable component in wheat bran, has its structure and function influenced by the extraction method. A two-step extraction method, involving sequential extraction with a dilute alkali followed by a concentrated alkali–urea mixture, was employed to extract AX from wheat bran. This approach aimed to obtain AX with a high phenolic acid content while achieving a relatively high extraction yield. The dilute alkali extraction could effectively retain the phenolic acid content in the AX extract (≤89 μg/g). However, its yield and sugar content were relatively low. In contrast, the alkali–urea extraction could achieve a relatively high yield (≤55%) and sugar content (≤75%). Different pretreatments (defatting, deproteinization, and delignification) were performed before extraction, causing significant changes to the chemical composition and cell wall structural characteristics of destarched wheat bran, which, in turn, affected the yield and composition of the AX extracts. Deproteinization effectively increased the sugar content, phenolic acid content, and overall yield of the extracts. Different pretreatment and extraction methods significantly affected the DPPH radical scavenging rate and Fe^2+^ chelating rate of the AX extracts but had little impact on the ABTS radical scavenging rate. The antioxidant activity of AX extracted using alkali–urea was unexpectedly higher than that extracted using a dilute alkali. This suggests that the antioxidant activity of AX does not entirely depend on its phenolic acid content but is influenced by various other factors.

## 1. Introduction

As a highly productive global crop, wheat supplies essential carbohydrates and proteins in human diets and is also a promising source of dietary fiber and other micronutrients [1,2]. In the wheat industry, approximately 150 million tons of wheat bran is generated per year [3], nearly 46% of which consists of non-starch polysaccharides, predominantly arabinoxylan (AX) [4]. AX is mainly composed of a main chain of β-(1,4)-d-xylan and side chains of arabinosyl and glucuronyl groups [5]. The majority of phenolic acids, mainly ferulic acid, present in wheat bran are ester-linked to the arabinosyl side groups of AX [5]. In recent decades, extensive research and practical experience have demonstrated that AX possesses multiple functional properties and bioactivities, for example, lowering cholesterol and regulating flora balance [6,7]. Therefore, how to efficiently separate AX from wheat bran and maximize its functionality has emerged as a key research topic.

However, most of the AX (ca. 95%) in wheat bran is water-unextractable because AX can bind to other cell wall components through covalent and non-covalent interactions [8,9]. In addition to cellulose and hemicellulose, wheat bran cell wall also contains components like lipids, proteins, and lignin, which may hinder the dissolution of AX. Rudjito et al. found that defatting improved the AX yield from subcritical water extraction and increased the ferulic acid content of AX extracts [10]. Similar results were found in alkaline extraction by Anderson et al., which was attributed to the removal of lipids that increased the alkali’s exposure to AX [4]. Cell wall proteins, like expansins, play a critical role in the crosslinking of cell wall polysaccharides, which may be another reason for the low extractability of AX [11]. Deproteinization is often employed in the pretreatment of wheat bran or post-treatment of polysaccharide extracts to improve their purity [4,12]. Lignin may be another factor that limits the dissolution of AX. AX can covalently link to lignin through the oxidative coupling of its side-chain ferulic groups. At present, extensive work is still needed to systematically compare the effect of these component on the extraction of AX and to clarify the necessity of removing them.

Several methods have been established to extract AX from wheat bran, including chemical extraction [13], enzymatic hydrolysis [5], hydrothermal extraction [14], and mechanically assisted extraction [8]. Among them, alkaline extraction primarily using NaOH, has become one of the most widely used methods for the extraction of wheat bran polysaccharides due to its advantages of high yield and good purity [15]. Jiang et al. optimized the conditions for ultrasonic/microwave-assisted alkaline extraction of corn AX and achieved a yield of AX of 27.78% and a total carbohydrate content of 78.05% [16]. The precooled NaOH solution, combined with urea, was also found to dissolve cellulose efficiently [17]. Cellulose, as a core component of the cell wall, dissolves in NaOH/urea solutions, potentially leading to the destruction of the cell wall and significant release of other polysaccharides. NaOH/urea solution was used to dissolve wheat bran, and 75.5% of AX was recovered from the resulting mixtures [18]. Although raising the alkali concentration can enhance the yield and purity of AX, the substantial loss of bound phenolic acids in AX due to saponification will decrease the functionality of AX. Paesani et al. extracted wheat bran non-starch polysaccharides under dilute alkali conditions (0.025–0.1 M NaOH) [19]. They found that extraction under 0.025 M NaOH achieved a high purity (88%) and antioxidant capacity, along with an acceptable yield (10%). Dilute alkaline solutions may avoid excessive hydrolysis of bound phenolic acids, highlighting their potential for extracting feruloyl AX.

To obtain feruloyl AX and maximize the polysaccharide yield from wheat bran, in this study, a two-step alkali extraction procedure was proposed. First, feruloyl AX was extracted using the dilute NaOH solution. Subsequently, a concentrated NaOH–urea was employed to extract the residual AX. Prior to extraction, pretreatments, including defatting, deproteinization, and delignification, were compared. The chemical composition and structural properties of the pretreated wheat bran were analyzed. Additionally, the extraction yield, composition, and antioxidant capacity of the AX fractions were evaluated.

## 2. Material and Methods

### 2.1. Materials

Wheat bran was provided by Shandong Fushikang Industry and Trade Group. Thermostable α-amylase (Termamyl SC DS), glucoamylase (Suhong GA III 2X), and Alcalase 2.4 L FG (food grade) were purchased from Novozymes (Beijing, China). Concentrated sulfuric acid and iodine were purchased from Sinopharm Chemical Reagent Co., Ltd. (Shanghai, China). Absolute ethanol, petroleum ether, glacial acetic acid, sodium hydroxide, copper sulfate, and urea were all purchased from Tianjin Kaitong Chemical Reagent Co., Ltd. (Tianjin, China). Total starch assay kits, D-Xylose (purity ≥ 99.8%), L-Arabinose (purity ≥ 99%), D-Galactose (purity ≥ 99%), D-Glucose (purity ≥ 99.8%), and gallic acid were purchased from Beijing Solarbio Science & Technology Co., Ltd. (Beijing, China). Sodium chlorite, ferulic acid, sinapic acid, p-coumaric acid, 3,4,5-trimethoxycinnamic acid, DPPH, and ABTS were all purchased from Shanghai Yuanye Biotechnology Co., Ltd. (Shanghai, China). Reduced iron powder, ferrozine, and FeCl_2_ were purchased from Shanghai Aladdin Biochemical Technology Co., Ltd. (Shanghai, China).

### 2.2. Pretreatment of Wheat Bran

**Figure 1:**
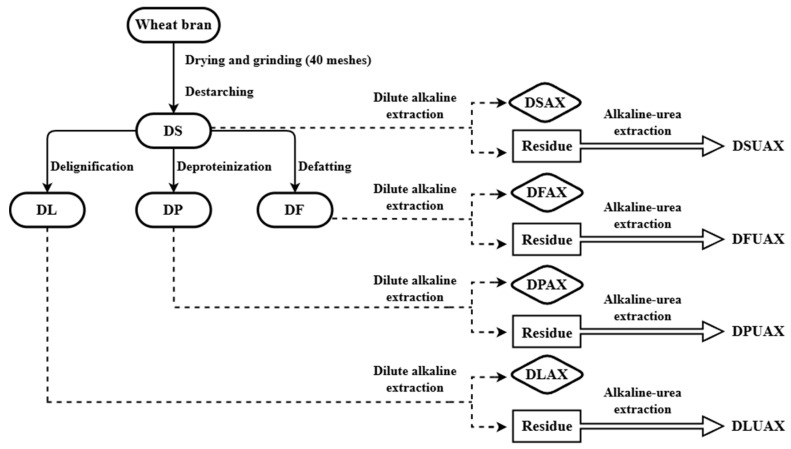
Procedure of wheat bran pretreatment and arabinoxylan extraction.

The wheat bran pretreatment procedure is presented in Figure 1. Wheat bran was dried and ground (40 meshes) before a destarching process. The destarching process was based on a previous study with slight modifications [20]. The ground wheat bran was suspended in water (1:10, *w*/*v*), the pH of the suspension was adjusted to 7.0 before preheating to 90 °C. Thermostable α-amylase and glucoamylase were added to the suspension and were incubated for 12 h with continuous stirring (5000 rpm). Iodine solution (0.5%) was used to indicate the removal of starch. The destarched wheat bran (referred to as “DS”) was recovered via successive centrifugation (4000 r/min, 15 min) and washed three times.

The destarched wheat bran (DS) was defatted according to the method of Hromádková et al. [21]. Briefly, 5 g of the sample was extracted with 80 mL of petroleum ether using a Soxhlet extractor. The extraction was performed at 135 °C for 120 min. The defatted wheat bran (referred to as DF) was air-dried in a fume hood.

Deproteinization of the DS was performed following the method of Anderson et al. [4]. The sample was suspended in deionized water (1:10, *w*/*v*) at 50 ℃, and the pH was adjusted to 7.0. Alcalase (2.4 L FG, 8424 U/g wheat bran) was added and was incubated for 3 h before inactivation in boiling water bath for 5 min.

Delignification of the DS was performed following the method of Rafidah et al. [22]. The sample was suspended in 0.2 M HCl (1:10, *w*/*v*), and an equal volume of 20% sodium chlorite solution was added slowly. The mixture was stirred at room temperature (5000 rpm) for 12 h before adjusting the pH to 7.0 to terminate the reaction. The delignified wheat bran (referred to as DL) was recovered via successive centrifugation (4000 r/min, 15 min) and washed three times.

### 2.3. Extraction of Arabinoxylan from Pretreated Wheat Bran

A two-step alkali extraction procedure was proposed, as shown in Figure 1. Wheat bran, whether pretreated or not, was first extracted with a dilute alkali solution. The resulting residue was then extracted with a urea–high-concentration alkali solution.

The dilute alkaline extraction was performed as described by Wang et al. with some modifications [13]. The sample was suspended in 0.1% NaOH solution (1:10 *w*/*v*) and incubated at 60 °C for 4 h with continuous stirring (5000 rpm). The residue was recovered by centrifugation (4000 r/min, 15 min) and washed with water twice. Before freeze-drying, the residue was adjusted to a pH of 7.0. The supernatant and wash water were combined, adjusted to a pH of 5.0, and centrifuged to remove proteins. The supernatant was then adjusted to a pH of 7.0 and mixed with three volumes of absolute ethanol for precipitation at 4 °C overnight. The precipitate (AX) was washed with three volumes of 80% ethanol and then freeze-dried. The AXs extracted from the destarched, defatted, deproteinized, and delignified wheat bran were referred to as DSAX, DFAX, DPAX, and DLAX, respectively.

The alkaline–urea extraction was performed as described by Yang et al. with minor modifications [23]. A solution containing 7% NaOH and 12% urea was precooled to −12.6 °C until ice crystals formed. The residue from the dilute alkali extraction was added to the precooled solution at a solid–liquid ratio of 1:20. The mixture was stirred at 25 °C (5000 rpm) for 12 h. The AXs and extraction residues were recovered using the same procedure as described for the dilute alkaline extraction. The AXs extracted from the destarched, defatted, deproteinized, and delignified wheat bran were referred to as DSUAX, DFUAX, DPUAX, and DLUAX, respectively.

### 2.4. Major Chemical Composition of Pretreated Wheat Brans

The determination of crude fat content was performed following the gravimetric method described in AOAC 954.02 [24]. The determination of protein content was performed using the Kjeldahl nitrogen determination method in AOAC 954.01, with a conversion factor of 6.25 used for calculation [25]. The determination of starch content was carried out using a total starch assay kit. The determination of cellulose, hemicellulose, and lignin content was performed following the NREL protocol [26]. A total of 300 mg of wheat bran sample was hydrolyzed with the addition of 3 mL sulfuric acid (72% *w*/*w*) at 30 °C for 1 h. The mixture was diluted with 84.0 mL of deionized water, sealed, and hydrolyzed in an autoclave at 121 °C for 2 h. The hydrolysate was filtered with a fritted crucible. The lignin content was determined gravimetrically based on the hydrolytic residue. The filtered hydrolysate was neutralized with calcium carbonate and analyzed using high-performance anion exchange chromatography (HPAEC) with a pulsed amperometric detector (Dionex ICS-5000+, Thermo Fisher Scientific, Waltham, MA, USA) equipped with a CarboPac PA20 analytical column (3 mm × 150 mm) [27].

### 2.5. Characterization of Pretreated Wheat Brans

The surface morphology of pretreated wheat bran was examined using a scanning electron microscope (Hitachi Regulus 8100, Tokyo, Japan) with an accelerating voltage of 8.0 kV. The particle size was measured using a laser particle size analyzer Haixinrui HL3100, Beijing, China) in a volume mode. The particle size distribution was expressed as the percentage of particles at different values of the particle diameter (D_50_), from which the median particle size diameter (D_50_) was obtained.

X-ray diffraction (XRD) was conducted using a Smartlab SE X-ray diffractometer (Rigaku, Tokyo, Japan). The XRD spectrum was scanned at 40 kV and 15 mA using a nickel-filtered Cu-Kα radiation source. The scanning range and scanning speed were 5°–40° and 2°/min, respectively.

Thermogravimetric analysis (TGA) was performed on an STA 449 F3 Jupiter thermal analyzer (Netzsch, Selb, Germany). A 3 mg sample was loaded into an alumina crucible surrounded by a nitrogen atmosphere maintained at a flow rate of 50 mL/min. The temperature was ramped up from 30 °C to 600 °C at a rate of 10 °C/min.

Fourier transform infrared (FTIR) spectra were recorded using a Nicolet iS10 FTIR Spectrometer (Thermo Fisher Scientific, USA). The sample was dispersed in KBr (1:80 *w*/*w*), ground, and pressed into a tablet. The scan range was 4000–400 cm^−1^ with a resolution of 4 cm^−1^ and 32 scans.

### 2.6. Characterization of Arabinoxylan Extracts

The extraction yield of arabinoxylan was calculated as the ratio of the freeze-dried arabinoxylan extract mass to the raw material mass. The monosaccharide composition of the arabinoxylan extract was determined according to the NREL laboratory analytical procedure with some modifications [26]. The acid hydrolysis procedure was scaled down to one-tenth of the original NREL procedure. The resulting hydrolysate was analyzed using high-performance anion exchange chromatography (HPAEC) as previously described [27]. UV–vis spectrum analysis was conducted according to Liu et al. [28]. The absorption spectrum of a 1 mg/mL sample solution was measured using a UV–vis spectrophotometer (T700AS, Puxi General Instrument, Beijing, China) with a scan range of 190–400 nm.

The analysis of phenolic acid content in arabinoxylan extracts was according to Yilmaz-Turan et al. [29]. A 20 mg sample was dissolved in 10 mL NaOH (2 M) and saponified at 35 °C for 2 h in the dark. After the solution was acidified to a pH of 2 with HCl, the phenolic acids were extracted with 3 times the volume of ethyl acetate, dried using a nitrogen stream, and redissolved in methanol. A high-performance liquid chromatography equipped with a C18 column (Inertsil ODS-SP 5 µm, 250 × 4.6 mm, Agilent Technologies) and time reaction detector (detection wavelength, 325 nm). The column temperature was 25 °C, the flow rate was 0.8 mL/min, and the injection volume was 20 μL. Two mobile phases were used: 0.1% aqueous formic acid solution (mobile phase A), 80% acetonitrile, and 10% methanol solution (mobile phase B). The elution procedures were 5–15% B (25 min), 15–22% B (17 min), 22–36% B (28 min), 36–5% B (5 min), and 5% B (5 min). Phenolic acid quantification was calibrated using a 1.0 mg/mL standard solution of ferulic acid, a 0.5 mg/mL standard solution of coumaric acid, and sinapic acid. All measurements were repeated three times.

The 1,1-diphenyl-2-picrylhydrazyl (DPPH), 2,2′-azino-bis(3-ethylbenzothiazoline-6-sulfonic acid) (ABTS) radical scavenging rate, and ferrous ions chelating capacity [30,31] were determined to evaluate the antioxidant activity of AX extracts. For the DPPH assay, 100 μL of the sample solution (1.25 mg/mL) was mixed with 100 μL of DPPH reagent (40 mg/L in 50% aqueous ethanol) and incubated at room temperature in the dark for 60 min. The absorbance of the mixture was measured at 517 nm using a microplate reader (BioTek Epoch 2, Winooski, VT, USA). For the ABTS assay, an ABTS stock solution was prepared by mixing 10 mL of a 7 mM ABTS solution and 10 mL of a 2.45 mM potassium persulfate solution. Before use, the ABTS stock solution was diluted with 50% aqueous ethanol until the absorbance at 734 nm was about 0.7. Then, 100 μL of the sample solution (0.5 mg/mL) was mixed with 100 μL of the diluted ABTS solution and incubated at room temperature in the dark for 30 min. The absorbance of the mixture was measured at 734 nm using a microplate reader. For the ferrous ion chelating capacity assay, 30 μL of the sample solution (1.25 mg/mL) was mixed with 70 μL of FeCl_2_ solution (0.107 mM) and 100 μL of ferrozine solution (0.75 mM), and incubated at room temperature for 10 min. The absorbance of the mixture was measured at 562 nm using a microplate reader. The DPPH, ABTS radicals scavenging rate, and Fe^2+^ chelating rate were calculated as follows:

(1) $$Scavenging/chelating\;rate\;\left(\%\right)=\left(1-\frac{A_{1}-A_{2}}{A_{0}}\right)\times 100$$

where *A*_0_ is the absorbance of the reagents, *A*_1_ is the absorbance of the incubated sample—reagent mixture, and *A*_2_ is the absorbance of the sample solution with reagents.

### 2.7. Statistical Analysis

All the quantitative experiments were performed in triplicates, and the results are expressed as mean values ± standard deviations. One-way ANOVA and Duncan’s multiple range test were performed using IBM SPSS Statistics 22.0 software to determine the significant difference (*p* < 0.05). Results labeled with the same letter indicate no significant differences.

## 3. Results and Discussion

### 3.1. Characterization of Different Pretreated Wheat Brans

Wheat bran was first destarched, and the destarched wheat bran was divided into four portions for defatting, deproteinizing, and delignifying, respectively (Figure 1). The contents of starch, fat, protein, and lignin in raw and pretreated wheat bran are shown in Table 1. The raw wheat bran used in this study had a high starch content (47%) and a relatively high protein content (16%). After the destarching process (DS), the starch content was reduced to 5%, while the protein content slightly increased. The destarching process removed 48.6% of the original wheat bran mass, primarily starch. Considering mass balance, besides starch, the destarching process also removed an amount of fat, protein, and water-soluble substances. The defatting process (DF) effectively removed liposoluble components from wheat bran, resulting in a significant reduction in fat content. A portion of lignin was also dissolved and removed during DF. The deproteinization process (DP) removed 74% of the protein content, with significant reductions in starch and fat also observed. Acidic sodium chlorite, an effective oxidative method of lignin removal with minimal impact on structural carbohydrates [32], was used to delignify the destarched wheat bran (DL). Compared with the DS sample, a 27% reduction in lignin was observed in the DL sample. The delignification process also caused a significant loss of fat and protein due to its strong oxidative effect. In addition, the DP and DL processes slightly altered the sugar composition of hemicellulose, as the A/X ratio increased from 0.9 to 1.1. This may indicate that the protein and lignin, which are closely linked to hemicellulose, were degraded by the DP and DL process, causing partial removal of some hemicellulose fractions.

**Figure 2:**
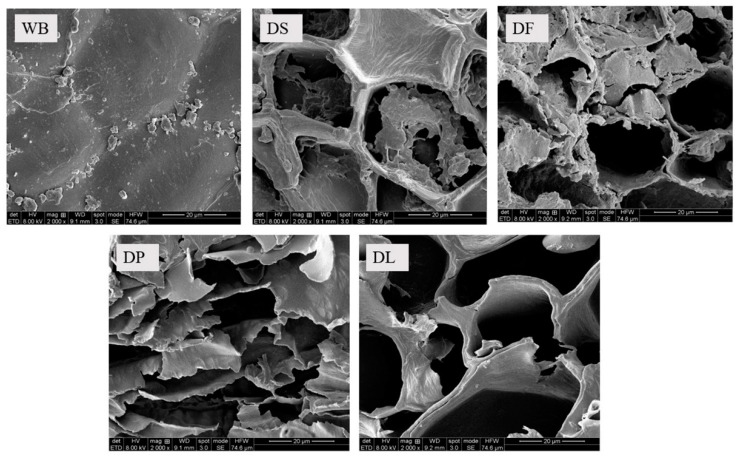
Surface morphology of the untreated and pretreated wheat bran samples. WB is untreated wheat bran, DS is destarched wheat bran, DF is defatted wheat bran, DP is deproteinized wheat bran, and DL is delignified wheat bran.

The surface morphology of the pretreated wheat bran samples was observed using SEM (Figure 2). The SEM images reveal the cell wall structure and cell contents. The untreated wheat bran (WB) showed an intact cell wall, with starch granules and protein aggregates adhering to its surface. The destarched wheat bran (DS) displayed a clearer skeleton of cell wall, as the starch granules and water-soluble components were removed. However, the integrity of the cell wall remained unaffected.

Both the defatting and deproteinization processes caused obvious damage to the cell wall structure. The cell wall of the defatted wheat bran (DF) exhibited partial fractures and deformation, while the deproteinized wheat bran (DP) showed a collapsed cell wall structure. In contrast, the delignified wheat bran (DL) maintained a relatively intact cell wall structure, indicating that the delignification process caused comparatively minor damage to the cell wall.

**Figure 3:**
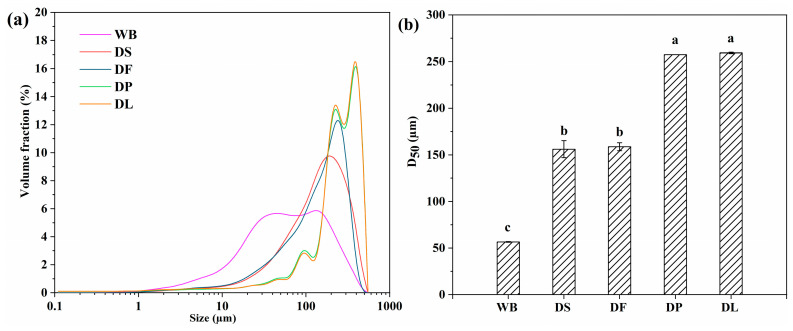
Particle size distribution (**a**) and median size (**b**) of the untreated and pretreated wheat bran samples. WB is untreated wheat bran, DS is destarched wheat bran, DF is defatted wheat bran, DP is deproteinized wheat bran, and DL is delignified wheat bran. Different letters (a–c) indicate significant differences. (*p* < 0.05).

The effect of pretreatment on the microstructure of wheat bran was also reflected in the particle size distribution. As shown in Figure 3, the particle size distribution of untreated wheat bran (WB) showed a broad bimodal shape, indicating high diversity in particle sizes. After the destarching process, the particle size distribution became more normalized, which was attributed to the removal of small particles, including starch granules, proteins, and other soluble components. The defatting process (DF) resulted in a more uniform particle size compared to the DS process, likely due to the removal of liposoluble components. Similar to the previous morphological results, the deproteinized sample (DP) and the delignified sample (DL) exhibited similar particle size distributions. Both displayed a narrow bimodal shape, with a new peak representing larger particles appearing adjacent to the previously existing peak. These observations indicate that all the pretreatments effectively removed small particles ranging from a few micrometers to several tens of micrometers. On the other hand, the pretreated wheat bran particles may be aggregated into larger particles due to the exposure of specific functional groups, such as hydroxyl and ionizable groups [33].

**Figure 4:**
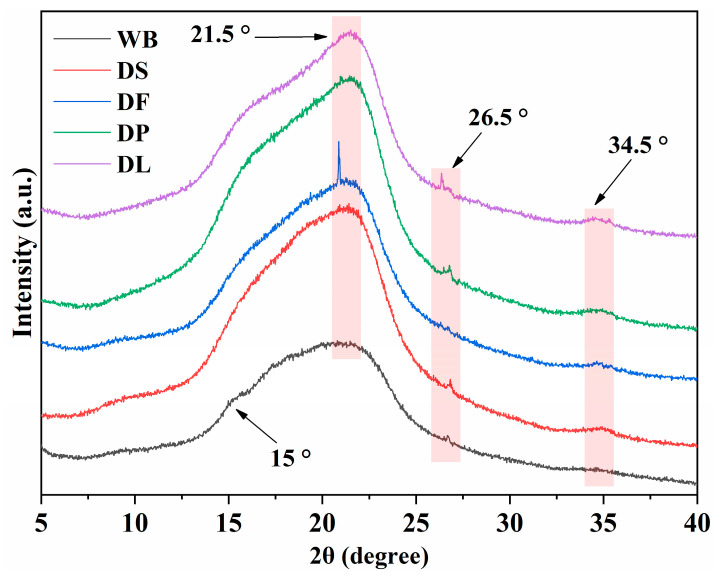
X-ray diffraction pattern of the untreated and pretreated wheat bran samples. WB is untreated wheat bran, DS is destarched wheat bran, DF is defatted wheat bran, DP is deproteinized wheat bran, and DL is delignified wheat bran.

XRD analysis was used to investigate the crystalline properties of different pretreated wheat brans (Figure 4). The crystalline substances in wheat bran are mainly cellulose and starch, while most of the other components are amorphous. The untreated wheat bran (WB) did not have any significant crystalline peaks except for a peak near 15°, probably due to the fact that pulverization destroys the crystalline structure [34]. After destarching, peaks at 21.5°, 26.5°, and 34.5° appeared. Subsequent pretreatments, including deproteinization and delignification, did not significantly alter the 2θ values of these peaks, although changes in peak intensity were observed. This indicates that the DP and DL pretreatment had little effects on the crystalline structure of the wheat bran. The removal of amorphous components enhanced the intensity of the XRD peak [35]. The peaks at 21.5° and 34.5° are attributed to the (110) and (004) planes of cellulose I crystals, respectively [36]. The peak at 26.5° disappeared in the defatted wheat bran (DF), which may be attributed to the removal of wax [37]. Since the pretreatments were conducted under mild conditions, no significant effects were observed on the robust structure of the crystalline structure of cellulose.

**Figure 5:**
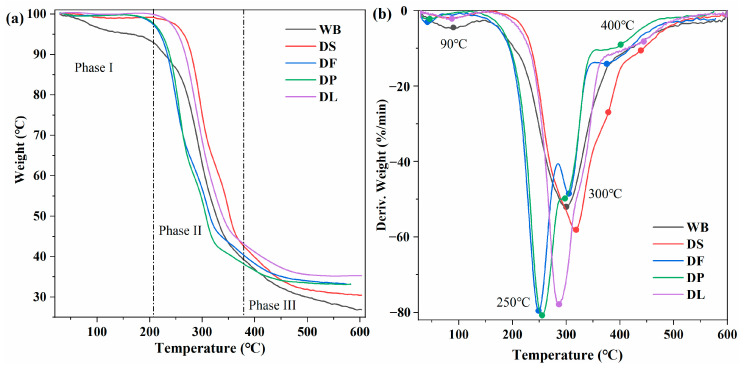
Thermogravimetric (**a**) and derivative thermogravimetric (**b**) curves of the untreated and pretreated wheat bran samples. WB is untreated wheat bran, DS is destarched wheat bran, DF is defatted wheat bran, DP is deproteinized wheat bran, and DL is delignified wheat bran.

Thermal stability, an important indicator of structural changes in the wheat bran cell wall, was analyzed using thermogravimetric analysis (Figure 5). The thermal degradation process could be clearly divided into three phases: the initial phase (30–200 °C), the main degradation phase (200–400 °C), and the high-temperature residual degradation phase (400–600 °C). This phenomenon is consistent with a previous report [38]. The initial phase is characterized by a relatively small mass reduction (approximately 5%) due to the evaporation of moisture. Compared with raw wheat bran, the pretreated wheat bran exhibited a slight reduction in moisture and volatile substances around 100 °C, indicating that the pretreatment removed bound water, free water, or other volatile compounds in the wheat bran. The main degradation phase (200–400 °C) is primarily attributed to the thermal degradation of cellulose and hemicellulose, the main components of the cell wall. During this phase, the decomposition of carbohydrates and the breakage of the hydrogen bond network resulted in a mass loss of approximately 60% [38], representing the primary thermal degradation process of the wheat bran samples. In the high-temperature residual degradation phase (400–600 °C), the mass loss is likely caused by the decomposition of highly structured components such as lignin or cellulose with a very dense structure [39].

The peak positions and intensities in the derivative thermogravimetry (DTG) curve of wheat bran reflect the pyrolysis temperatures and weight loss rates of its components, indirectly indicating their relative contents. The DTG curve of untreated wheat bran (WB) shows a broad peak at about 300 °C, attributed to the close connection and interaction between the many complex components of starch, cellulose, and proteins, resulting in a broadening of the peak shape after the degradation peaks overlap [40]. Following pretreatment, this broad peak gradually evolves into two more distinct peaks, corresponding to the removal of starch, fat, and protein, which highlights the pyrolysis characteristics of cellulose, hemicellulose, and lignin. Specifically, the DTG curves of defatted (DF) and deproteinized (DP) samples show clear pyrolysis peaks at 250 °C and 300 °C, associated with hemicellulose and cellulose, respectively. In this process, hemicellulose and cellulose undergo decarboxylation, oxidation, and decomposition, resulting in the breakage of side chains and main chains and the generation of a series of hydrocarbons such as carbon monoxide and carbon dioxide, leading to the mass loss phenomenon [41]; while the delignification treatment significantly reduces the intensity of the pyrolysis peak of lignin near 450 °C [42]. These changes strongly prove that different pretreatments reduce the thermal stability of wheat bran, facilitating the extraction of polysaccharides.

**Figure 6:**
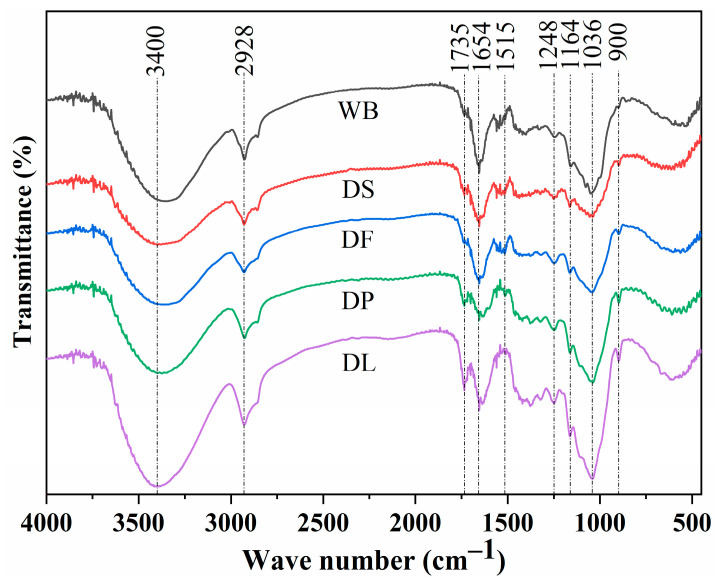
Fourier transform infrared spectra of the untreated and pretreated wheat bran samples. WB is untreated wheat bran, DS is destarched wheat bran, DF is defatted wheat bran, DP is deproteinized wheat bran, and DL is delignified wheat bran.

In Figure 6, the spectral range of 900–1200 cm^−1^ is the characteristic band of arabinoxylan [13]. In particular, the increased peak area at 1047 cm^−1^ (C-O stretching vibration on the glycosidic bond) confirms the effectiveness of defatting, deproteinizing, and delignifying treatments in improving the polysaccharide extraction rate [21]. Further analysis indicates that the diminished peaks at 1654 cm^−^¹, 1540 cm^−^¹, and 2900 cm^−^¹, as well as changes in the peak shape in Figure 6, are related to the decrease in protein and lipid content in wheat bran, respectively. These findings are consistent with the data in Table 1 [43]. Additionally, the strong and broad absorption peaks at 3300~3500 cm^−1^ are caused by the O-H stretching vibrations within or between the cellulose and hemicellulose molecules [13]. Compared with the DS sample, defatting, deproteinizing, and delignifying all promote an increase in peak area, indicating that more hydroxyl groups were exposed, which might have resulted from the broken glycosidic bonds [34].

In addition, the enhanced peak near 1735 cm^−1^ is related to carbonyl groups, directly confirming the presence of uronic acid [16]. This indicates that the uronic acid content in the DF, DP, and DL samples is higher compared to the DS sample. The peak near 1164 cm^−1^ indicates C-O stretching in cellulose, and delignification increases the relative proportion of cellulose. However, the reduction in the peak intensity at this position in the DF sample may result from the dissolution of some cellulose, likely caused by the disruption of its amorphous regions, crystallinity, and long-chain structure [44]. The weak absorption peak near 1500 cm^−1^ represents aromatic skeleton vibrations, likely originating from lignin and phenolic compounds. The weakened peak intensity at this position in the DL sample is attributed to the reduction of lignin and phenolic acid content caused by delignification pretreatment [45]. The weak absorption peak around 1735 cm^−1^ represents the stretching vibration of acetyl and uronic acid groups in hemicellulose and/or the ester linkage of ferulic acid and p-coumaric acid carboxyl groups. Considering the increased peak intensity in the DL sample, it is speculated that it is due to the C=O groups generated by the oxidation of C-OH groups during the delignification process, which is consistent with the findings of Debiagi et al. [42]. Thus, the effect of pretreatment on wheat bran is reflected in the significant differences in the content of specific components and the interaction between molecules.

### 3.2. Extraction Yield and Sugar Composition of Arabinoxylan Extracts

**Figure 7:**
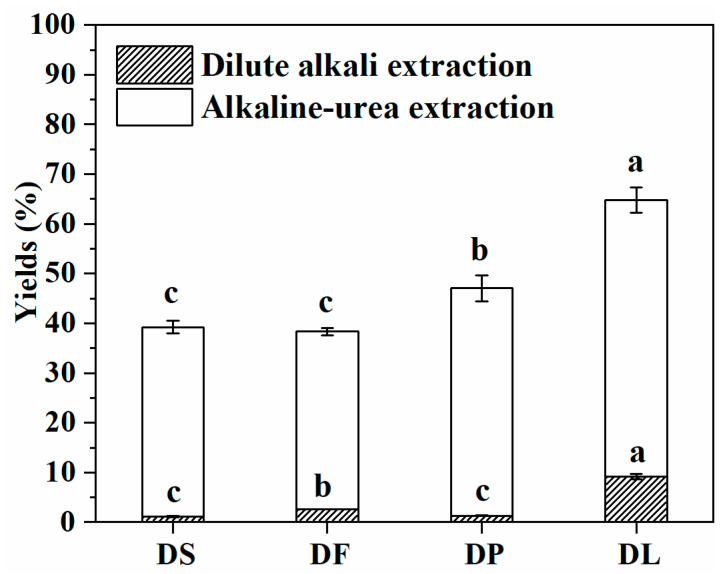
The extraction yields of arabinoxylan from the pretreated wheat bran samples. WB is untreated wheat bran, DS is destarched wheat bran, DF is defatted wheat bran, DP is deproteinized wheat bran, and DL is delignified wheat bran. Different letters (a–c) indicate significant differences. (*p* < 0.05).

The results in Figure 7 show a significant improvement in the yield of the alkali–urea extraction compared to dilute alkali extraction. This significant difference is mainly attributed to the effect of the elevated alkali concentration [18,19]. Specifically, the high efficiency of the urea/concentrated alkali extraction method can be explained from two perspectives. First, extending the extraction time allows for the full infiltration and diffusion of raw materials, thereby improving the accessibility and dissolution efficiency of polysaccharides. Second, urea can disrupt the hydrogen bonds between polysaccharide molecules, enhancing the solubility of polysaccharide in an alkali [18], which further facilitates the complete release of polysaccharides.

The yields of dilute alkali extraction from different pretreated samples range between 1% and 10%, which aligns with the findings of Mouzakitis et al. [8] (yielding 5.40% under the conditions of 0.1 M NaOH, 60 °C, and 4 h). For alkali–urea extraction, we found that deproteinization (DP) and delignification (DL) pretreatments significantly improved the extraction efficiency. Among them, DL exhibited the best performance, with its yield increasing by 17.54% compared to the destarch (DS) pretreatment, followed by DP, which showed a yield increase of 7.6%. However, defatting treatment did not significantly promote the yield of the alkali–urea extraction. This may be because lipids are prone to saponification and dissolution in a concentrated alkali environment, making them less of an obstacle to extraction. In contrast, the complex carbohydrate–protein conjugate structure in wheat bran is considered one of the key factors hindering the efficient extraction of polysaccharides [18], providing an important clue for the subsequent optimization of the polysaccharide extraction process.

The monosaccharide composition and protein content of the arabinoxylan extracts from different pretreated wheat bran by dilute alkali extraction and alkali–urea extraction are presented in Table 2. The major monosaccharides of all extracts were xylose and arabinose, which are the predominant sugars of arabinoxylan. Moreover, some glucose and galactose were detected in the extracts. The glucose may be derived from β-glucan or the side chains of AX. The galactose was also found on the side chains of AX [46]. The extraction method and pretreatment both had effects on the monosaccharide composition. The extracts from dilute alkali extraction showed a relatively lower total sugar content compared to the alkali–urea extraction, indicating that a dilute alkali to AX has lower selectivity for AX than alkali–urea extraction. This may be due to the limited destruction of AX crosslinking in a dilute alkali, while other components in wheat bran are more soluble in it. On the other hand, dilute alkali extraction removed more non-sugar components from the wheat bran, thus reducing the chance of these components being mixed into the extracts of alkali–urea extracts.

Among different pretreatment methods, the extracts obtained from the deproteinized wheat bran (DPAX and DPUAX) showed the highest total sugar content, implying that the DP process may remove the structural proteins in cell walls, thus promoting the solubilization of polysaccharides. The extracts from defatted (DFAX and DFUAX) and delignified (DLAX and DLUAX) wheat brans showed a lower content of total sugar, compared to the extracts from destarched wheat bran. According to previous analysis, both DF and DL pretreatments had an impact on the structure of wheat bran. Moreover, the yields of DFAX, DLAX, and DLUAX were higher than the corresponding DSAX and DSUAX. Combining these results, we speculate that DF and DL may break the structure of non-sugar components such as lignin and wax, and let them co-dissolve with polysaccharides [47], resulting in a decrease in the sugar content of polysaccharide extracts. In addition, the relative trend of the protein content of different pretreated extracts was similar to that of pretreated wheat bran.

The A/X ratio reflects the degree of substitution of arabinosyl on the xylan backbone in AX [14]. All the extracts except DPUAX had similar A/X ratios (0.52–0.63), while DPUAX had a higher A/X ratio of 0.82. This suggests DPUAX contained more branched AX fractions, which may result from highly cross-linked AX components dissolved due to the disruption of structural proteins.

**Figure 8:**
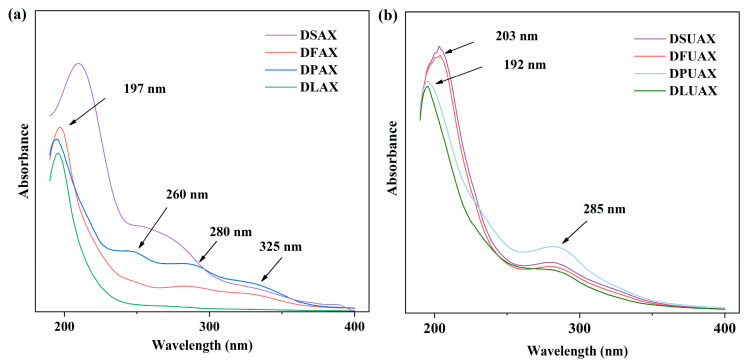
UV–vis spectra of arabinoxylans extracted by dilute alkali (**a**) and concentrated alkali–urea mixture (**b**).

Figure 8a,b show the UV–vis absorption spectra of AX extracted from different types of pretreated wheat bran by dilute alkali and alkali–urea extractions, respectively. All the spectra exhibit a strong absorption peak near 200 nm, which can be attributed to n → π* transitions of aldehydes or ketones in polysaccharides [28]. The absorption peak at 250–350 nm may reflect the presence of proteins, phenolics, and other chromophoric impurities [48,49]. It is worth noting that different pretreatments caused significant changes in these absorption peaks of the dilute alkali extracts. Defatting (DFAX) and delignification (DLAX) seemed more efficient at removing components with chromophores. In comparison, all the UV spectra of AX extracts obtained via alkali–urea extraction were similar, showing absorption peaks near 200 nm and at 285 nm. The absorption peak at 285 nm may be ascribed to proteins or free phenolic acids, while the peaks at 320–340 nm observed in dilute alkali extracts may reflect the presence of bound phenolic acids [21].

### 3.3. Phenolic Acid Composition and Antioxidant Capacity of Arabinoxylan Extracts

The phenolic acid content of different arabinoxylan extracts was analyzed by reversed-phase HPLC. Ferulic acid, sinapic acid, and p-coumaric acid were detected and quantified (Table 3). Ferulic acid is the major phenolic acid present in wheat bran, while other phenolic acids were reported in a minor amount [30]. Phenolic acids are covalently bound to AX through ester bonds, so they can be extracted synchronously with AX. However, different extraction methods can more or less affect the content of bound phenolic acids in AX extracts. Since ester bonds are vulnerable to an alkali, it is conventionally believed that the loss of phenolic acids during alkali extraction is significant. Here in this research, the phenolic acid content in the AX extracted with a dilute alkali was notably higher than that obtained with alkali–urea extraction, except for the group pretreated with acidic chlorite. A dilute alkali cannot completely break the cross-linking structures between polysaccharides, and its hydrolysis efficiency of phenolic acid ester bonds is also relatively low [13]. Therefore, the AX extracted by a dilute alkali can effectively retain the bound phenolic acids. For DSAX, DFAX, and DPAX, ferulic acid constituted approximately 97% of all phenolic acids.

Different pretreatment methods showed significant effects on the content of phenolic acids in the AX extracts by dilute alkali extraction. When compared with the sugar composition (Table 2), it was found that the phenolic acid contents showed a similar trend to that of the total sugar content. This suggests that the phenolic acids were extracted together with the AX because they are covalently bonded. The deproteinization process combined with dilute alkaline extraction (DPAX) showed a notable recovery of phenolic acids. For DLAX and DLUAX, the delignification pretreatment using acidic chlorite was expected to eliminate all lignin-like components, including phenolic acids. This was confirmed as their phenolic acid content was the lowest.

The scavenging rate against DPPH and ABTS radicals, as well as the Fe^2+^ chelating rate, was analyzed to investigate the in vitro antioxidant capacity of the AX extracts (Table 4). For different pretreatment and extraction methods, the DPPH radical scavenging rate and Fe^2+^ chelating rate showed more pronounced differences than the ABTS radical scavenging rate.

The DPPH radical scavenging rate of AX extracts from wheat bran pretreated by dilute alkali extraction showed significant differences. DPAX had the highest scavenging rate (59.62%), followed by DSAX (43.43%), while DFAX and DLAX had much lower scavenging rates. The DPPH assay is more sensitive to hydrophobic antioxidants. The low DPPH radical scavenging rate of DFAX and DLAX may be due to the removal of hydrophobic antioxidant components or groups. The AX extracts from alkali–urea extraction had higher DPPH radical scavenging rates than the corresponding extracts from dilute alkali extraction. The DPPH radical scavenging rates of AX extracts from alkali–urea extraction were consistently higher than those from dilute alkali extraction. This may also be due to the presence of hydrophobic antioxidant structures in the polysaccharide molecules of the AX extracts from alkali–urea extraction.

The Fe^2+^ chelating rate of DSAX was much higher than that of other AX extracts from dilute alkali extraction; this may be the result from the retention of chelating agents such as phytic acid and phospholipids. AX extracts from alkali–urea extraction also showed a higher Fe^2+^ chelating rate. Under higher alkali concentrations, AX fractions with more uronic acid groups are more likely to solubilize and may contribute to the higher chelating capacity.

The free radical scavenging rates and phenolic acid content (Table 3) were weakly correlated, indicating that the antioxidant capacity of AX extracted by alkali did not primarily depend on its phenolic acid content. Similar results were reported by Yilmaz-Turan et al. [29], who suggested that the state of phenolic acids (bound or free) or the presence of other antioxidants may significantly affect the antioxidant properties of AX extracts. Furthermore, the antioxidant properties of polysaccharides are also influenced by a combination of complex factors such as uronic acid content, molecular weight, monosaccharide composition, and chain conformation [50].

## 4. Conclusions

A two-step alkali extraction process, involving sequential extraction with dilute alkali followed by an alkali–urea mixture, was proposed to prepare AX from wheat bran. The effect of the pretreatment on removing different non-sugar components from wheat bran was investigated. This two-step alkali extraction process resulted in AX with a relatively high phenolic acid content (≤89 μg/g) and a relatively high overall yield of AX (≤65%). Most AX extracted by dilute alkali had a higher phenolic acid content than that extracted by alkali–urea, yet its yield and sugar content were much lower. Further pretreatment through defatting, deproteinization, or delignification led to variations in the chemical and structural characteristics of the de-starched wheat bran, thereby influencing the dissolution of AX from the cell wall. Different pretreatment methods influenced the AX extract composition obtained by dilute alkali extraction. Deproteinization increased the sugar and phenolic acid content and overall yield of the AX extracts. Unexpectedly, the antioxidant capacity of AX extracts did not mainly depend on its phenolic acid content but might be affected by a variety of other factors.

This study indicated that by combining appropriate pretreatment and extraction strategies, AX extracts with enhanced functionality could be obtained. Future research is necessary to further explore the mechanism of action of deproteinization on the cell wall structure, optimize the extraction strategy, and clarify the synergistic effects of AX’s structural factors on its antioxidant capacity. In the future, this study encourages further exploration of the deep-seated effects of different pretreatment conditions on the functional properties of wheat bran, taking this as an opportunity to develop high-value-added products based on wheat bran and to promote the development of the grain processing industry towards a high-end status and refinement. Through continuous scientific research and technological innovation, wheat bran, a traditional agricultural by-product, will be revitalized and bring revolutionary changes to many fields such as food, medicine, and health care products.

## Figures and Tables

**Table 1:** Chemical composition (wt%) of the untreated and pretreated wheat bran samples.

Constituents	WB	DS	DF	DP	DL
Starch	50.22 ± 0.08 ^a^	5.66 ± 0.16 ^b^	3.16 ± 0.40 ^d^	2.72 ± 0.12 ^d^	4.09 ± 0.18 ^c^
Fat	4.32 ± 0.05 ^a^	4.26 ± 0.48 ^a^	0.85 ± 0.16 ^c^	1.90 ± 0.06 ^b^	1.19 ± 0.03 ^c^
Protein	17.30 ± 0.09 ^c^	18.91 ± 0.10 ^b^	20.48 ± 0.11 ^a^	4.89 ± 0.03 ^d^	4.29 ± 0.03 ^e^
Lignin	8.33 ± 0.13 ^d^	15.08 ± 0.23 ^a^	12.25 ± 0.13 ^b^	15.54 ± 0.17 ^a^	11.04 ± 0.26 ^c^
Cellulose	11.94 ± 0.21 ^c^	20.68 ± 0.44 ^b^	21.35 ± 0.35 ^b^	24.15 ± 0.62 ^a^	25.02 ± 0.69 ^a^
Hemicellulosic sugar composition					
Arabinose	8.87 ± 0.11 ^d^	16.06 ± 0.20 ^c^	15.75 ± 0.04 ^c^	23.03 ± 0.43 ^a^	22.10 ± 0.13 ^b^
Xylose	9.62 ± 0.08 ^d^	17.42 ± 0.14 ^c^	16.50 ± 0.17 ^c^	20.44 ± 0.53 ^a^	18.74 ± 0.67 ^b^
Galactose	4.07 ± 0.04 ^d^	7.37 ± 0.07 ^c^	10.39 ± 0.50 ^a^	10.93 ± 0.04 ^a^	9.13 ± 0.95 ^b^
A/X ratio *	0.92	0.92	0.95	1.13	1.18

Expressed as mean ± standard deviation (*n* = 3). Different letters within the same row indicate significant differences (*p* < 0.05). * A/X ratio is the ratio between arabinose and xylose.

**Table 2:** Sugar composition and protein content of the arabinoxylan extracts.

Extracts	Sugar Composition				Total Sugar (%)	A/X Ratio	Protein (%)
	Arabinose (%)	Galactose (%)	Glucose (%)	Xylose (%)			
DSAX	10.34 ± 1.23 ^e^	0.79 ± 0.08 ^e^	4.94 ± 0.88 ^de^	17.12 ± 2.65 ^e^	33.20 ± 2.42 ^d^	0.59	9.24 ± 0.21 ^a^
DFAX	6.03 ± 0.21 ^f^	0.62 ± 0.01 ^f^	3.62 ± 0.12 ^e^	11.31 ± 0.15 ^f^	21.58 ± 0.24 ^e^	0.52	6.97 ± 0.15 ^c^
DPAX	16.10 ± 0.53 ^d^	1.38 ± 0.11 ^cd^	6.28 ± 0.30 ^d^	27.69 ± 1.25 ^d^	51.46 ± 1.10 ^c^	0.57	4.62 ± 0.05 ^d^
DLAX	3.01 ± 0.21 ^g^	0.18 ± 0.01 ^g^	1.16 ± 0.12 ^f^	4.70 ± 0.45 ^g^	9.04 ± 0.39 ^f^	0.63	3.78 ± 0.12 ^e^
DSUAX	20.77 ± 0.09 ^b^	1.64 ± 0.06 ^b^	14.69 ± 0.82 ^b^	37.95 ± 0.43 ^a^	75.04 ± 0.70 ^a^	0.53	7.89 ± 0.08 ^b^
DFUAX	18.54 ± 0.29 ^c^	1.30 ± 0.05 ^d^	12.56 ± 0.99 ^c^	32.60 ± 0.49 ^b^	65.02 ± 0.62 ^b^	0.56	6.90 ± 0.09 ^c^
DPUAX	25.14 ± 0.42 ^a^	2.05 ± 0.10 ^a^	17.83 ± 0.67 ^a^	29.93 ± 0.29 ^c^	74.95 ± 0.74 ^a^	0.82	3.98 ± 0.30 ^e^
DLUAX	20.75 ± 1.61 ^b^	1.56 ± 0.22 ^bc^	12.12 ± 1.67 ^c^	33.24 ± 1.16 ^b^	67.66 ± 2.33 ^b^	0.61	1.35 ± 0.10 ^f^

Expressed as mean ± standard deviation (*n* = 3). Different letters within the same column indicate significant differences (*p* < 0.05).

**Table 3:** Analysis of the phenolic acids of AX extracted by different alkalis.

Samples	Ferulic Acid (μg/g)	Sinapic Acid (μg/g)	P-Coumaric Acid (μg/g)	Total Phenolic Acid (μg/g)
DSAX	34.74 ± 2.83 ^b^	0.22 ± 0.05 ^b^	0.78 ± 0.11 ^b^	35.78 ± 0.09 ^b^
DFAX	32.22 ± 1.92 ^c^	0.18 ± 0.07 ^b^	0.90 ± 0.16 ^b^	33.37 ± 0.13 ^c^
DPAX	87.23 ± 1.63 ^a^	0.61 ± 0.12 ^a^	1.84 ± 0.05 ^a^	88.99 ± 0.71 ^a^
DLAX	0.50 ± 0.14 ^d^	0.05 ± 0.01 ^c^	0.47 ± 0.08 ^c^	0.86 ± 0.21 ^e^
DSUAX	1.86 ± 0.06 ^d^	0.20 ± 0.01 ^b^	0.53 ± 0.05 ^c^	2.40 ± 0.24 ^d^
DFUAX	1.51 ± 0.14 ^d^	0.17 ± 0.01 ^b^	0.42 ± 0.02 ^c^	1.96 ± 0.01 ^d^
DPUAX	1.34 ± 0.40 ^d^	0	0.47 ± 0.03 ^c^	1.86 ± 0.02 ^d^
DLUAX	0.78 ± 0.12 ^d^	0	0.42 ± 0.03 ^c^	1.11 ± 0.06 ^e^

Expressed as mean ± standard deviation (*n* = 3). Different letters within the same column indicate significant differences (*p* < 0.05).

**Table 4:** In vitro antioxidant activities of AX extracted by different alkalis.

Samples	DPPH Radical Scavenging Rate (%)	ABTS Radical Scavenging Rate (%)	Fe^2+^ Chelating Rate (%)
DSAX	43.43 ± 1.81 ^b^	24.64 ± 21.52 ^abc^	47.96 ± 0.68 ^a^
DFAX	26.50 ± 1.89 ^d^	21.52 ± 3.04 ^bcd^	9.81 ± 1.11 ^e^
DPAX	59.62 ± 0.26 ^a^	25.18 ± 1.24 ^ab^	13.41 ± 1.53 ^e^
DLAX	23.91 ± 1.43 ^d^	13.57 ± 1.75 ^e^	11.68 ± 1.18 ^e^
DSUAX	46.04 ± 0.62 ^b^	19.55 ± 2.63 ^d^	32.84 ± 1.77 ^b^
DFUAX	44.78 ± 4.18 ^b^	21.43 ± 1.08 ^bcd^	28.40 ± 0.86 ^c^
DPUAX	55.60 ± 2.69 ^a^	26.34 ± 0.58 ^a^	35.34 ± 3.12 ^b^
DLUAX	34.21 ± 0.36 ^c^	20.71 ± 1.43 ^cd^	18.13 ± 0.83 ^d^

DPPH radical scavenging activity (%) represents the scavenging rate of DPPH radicals obtained by 1.25 mg/mL polysaccharide solution. ABTS radical scavenging activity (%) represents the scavenging rate of ABTS radicals obtained by 0.5 mg/mL polysaccharide solution. Fe^2+^ chelating activity (%) represents the chelation rate of ferrous ions by a 1.25 mg/mL polysaccharide solution, expressed as mean ± standard deviation (*n* = 3). Different letters within the same column indicate significant differences (*p* < 0.05).

## Data Availability

The original contributions presented in the study are included in the article; further inquiries can be directed to the corresponding author.
